# Conversion to Graves disease from Hashimoto thyroiditis: a study of 24 patients

**DOI:** 10.20945/2359-3997000000086

**Published:** 2018-10-01

**Authors:** Beatriz Gonzalez-Aguilera, Daniela Betea, Laurence Lutteri, Etienne Cavalier, Vincent Geenen, Albert Beckers, Hernan Valdes-Socin

**Affiliations:** 1 University Hospital Juan Ramón Jiménez Quirónsalud Hospital Group Department of Endocrinology Sevilla Spain Department of Endocrinology, University Hospital Juan Ramón Jiménez (Huelva), Quirónsalud Hospital Group, Sevilla, Spain; 2 CHU Department of Endocrinology Liège Belgium Department of Endocrinology, CHU, Liège, Belgium; 3 CHU Department of Clinical Biology Liège Belgium Department of Clinical Biology, CHU, Liège, Belgium

**Keywords:** Hashimoto's thyroiditis, TBII, Graves hyperthyroidism, TSH receptor antibodies, autoimmune conversion

## Abstract

**Objective:**

The conversion of Hashimoto's thyroiditis (HT) to hyperthyroidism due to thyrotropin receptor antibodies is intriguing and considered rare. The contribution of TSH receptor blocking antibodies (TRAb), which may be stimulators (TSAb) or blockers (TBAb), is suspected. We describe clinical and biological variables in a series of patients switching from Hashimoto's thyroiditis to Grave's disease.

**Subjects and methods:**

Retrospective case study of 24 patients with Hashimoto's thyroiditis followed during 48 ± 36 months that developed later Graves’ disease (GD). These variables were analysed in the hypo and hyperthyroid phase: age, sex, initial TSH, free triiodothyronine (fT3), free thyroxine (fT4), anti-TPO, TBII antibodies, parietal cell autoantibodies, time between hypo and hyperthyroidism, thyroid volume and levothyroxine doses (LT).

**Results:**

In HT, mean TSH was 9.4 ± 26.1 UI/L and levothyroxine treatment was 66.2 ± 30.8 µg/day. The switch to GD was observed 38 ± 45 months after HT diagnosis. As expected, we found significant differences on TSH, FT3, FT4 and TBAb levels. Three out of 14 patients had parietal cell autoantibodies. In two of these three cases there was an *Helicobacter pylori* infection. There were no significant differences between HT and GD groups with respect to thyroid volume.

**Conclusions:**

To our knowledge, large series documenting the conversion of HT to GD are scarce. Although rare, this phenomenon should not be misdiagnosed. Suspicion should be raised whenever thyroxine posology must be tapered down during the follow-up of HT patients. Further immunological and genetic studies are needed to explain this unusual autoimmune change.

## INTRODUCTION

Hashimoto's thyroiditis (HT) and Graves’ disease (GD) are autoimmune thyroid disorders (AITDs) with different physiopathology, being traditionally regarded as two different disease entities. More recent views, in contrast, have considered the hypothesis that there might be a continuum between HT and GD (1,2). Recently, it has been sporadically reported that GD and HT may follow one another in the same individuals due to a sequential phenotypic conversion from GD to HT or *vice versa* (3,4).

The most common scenario is the evolution from GD into HT, whereas the switch from HT into GD seems to be less common. This is probably due to the lack of a critical mass of functioning thyroid tissue able to react to thyrotropin (TSH) receptor autoantibodies (TRABs) in individuals with long-standing HT (5).

**Figure 1:**
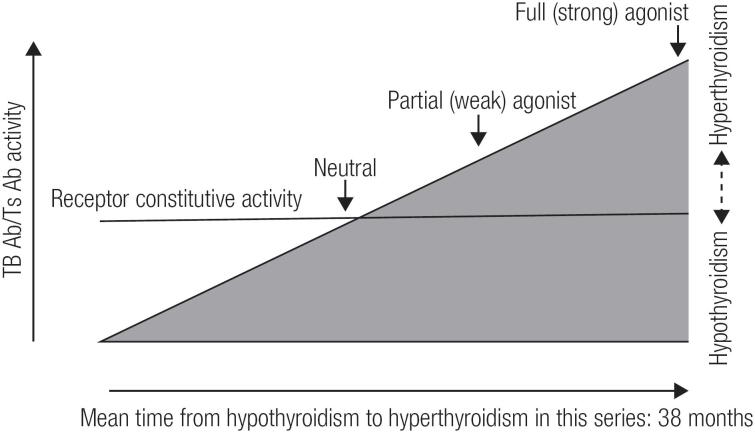
The figure describes the transition from hypothyroidism to hyperthyroidism during follow up in the same patient. The phenomenon is due to a shift in the concentrations and affinities of TBAb and TSAb. In our series the mean time for this switch was of 38 months. Thyrotropin (TSH) has a constitutive activity, as shown by the horizontal line through the wedge. TSHR ligand (TSH or TSAb) further increase receptor activity. An inverse agonist supresses constitutive activity, whereas a full agonist maximally activates the receptor. Ligands of intermediate activity are either neutral agonists or partial agonists or inverse agonists. Ligands can also be an antagonist depending on their relative affinities and binding sites. Therefore, TSAb beeing a partial agonist may also be an antagonist for TSH. If a serum displays both TSAb and TBAb activity, unless the former is very weak and the latter is very strong, it cannot be assumed that there are two separate antibodies. [Adapted from: McLachlan et al. (13)].

The phenomenon of the switch from Hashimoto's thyroiditis to hyperthyroidism and/or Graves’ ophthalmopathy is also known as ‘hypothyroid Graves’ disease’ being first described by Wyse and cols. in 1968 (6). Since the first description of these cases was given, the concept of hyperthyroidism as an essential component of Graves’ disease has been modified. For 50 years, several similar cases have been published. However, texts and medical books hardly refer to this phenomenon. Moreover, the pathogenic mechanism is not well known (6-11). One of the most accepted hypotheses is the potential contribution of blocking antibodies (TBAb) and TSH receptor stimulators (TSAb) (8-13). TSAb antibodies are responsible for Graves’ disease hyperthyroidism while TBAb antibodies are sometimes responsible for a pattern of hypothyroidism. The change of antibodies from TBAb to TSAb (or *vice versa*) occurs rarely in patients treated with levothyroxine for hypothyroidism or in patients with Graves’ disease treated with antithyroid drugs. These changes involve differences in the concentrations, affinities and potency of TSAb and TBAb. Thus, the increase of TSAb after treatment with levothyroxine may be sufficient to counteract the TBAb antibodies and result in hyperthyroidism (Figure 1). In the present study, we describe the clinical, immunological and biochemical characteristics of patients with HT, who later developed GD. We discuss our results and compare them with the available literature.

## MATERIALS AND METHODS

### Design

This study is an observational retrospective case study performed in the Centre Hospitalier Universitaire de Liège. Among nearly 2000 patients with HT followed up in our endocrine unit during the period 2000-2016, we identified a group of 24 patients. These patients represented 1.2% of the series. Moreover, the 24 patients performed a posterior switch to GD. All the data recorded at HT diagnosis and at GD presentation were anonymously reconstructed after informed consent, from patients’ files.

### Eligibility criteria

The inclusion criteria were as follows: males and females with HT and age ≥ 18. The criteria for the diagnosis of HT were: a) Elevated TSH levels > 4 mUI/L, the presence of serum thyroid peroxidase autoantibodies (TPOAbs) at titres above the upper limits of the reference ranges; b) hypoechogenic thyroid pattern at ultrasonography (US) consistent with AITD. The absence of Thyroid-Binding Inhibitory Immunoglobulin (TBII) was determined in only three patients.

The criteria for diagnosis of GD were as follows: a) elevated levels of free thyroxine (T4) and suppressed TSH; b) the presence of TBII; c) hypoechogenic thyroid pattern at US consistent with AITD; d) no tendency to spontaneous normalization of hyperthyroidism before methimazole treatment; and e) positive technecium^99^/iodine^123^ thyroid scintigraphy. The exclusion criteria were as follows: hypothyroidism due to other causes, and patients < 18 years.

### Assessment tools and variables

The following variables were collected: age, sex, TSH, free T3 and free T4 at the beginning of the study and at the moment of the switch to hyperthyroidism, anti-TPO and TBII antibody titres in the hypo and hyperthyroid phase, time evolution of hypothyroidism, time between hypo and hyperthyroidism, levothyroxine doses (LT) in both groups, presence or absence of Graves’ ophthalmopathy, positivity for anti-parietal cell antibodies and type of treatment for hyperthyroidism.

### Laboratory tests

Serum concentrations of TSH (normal range 0,27–4,2 mU/L), fT4 (normal range 11,6-21,9 pmol/L) and fT3 (normal range 3-6,8 pmol/L) were measured by electrochemiluminescence methods (using system Cobas e801, Roche^®^) (14-16).

Anti-TPO antibodies were measured by electrochemiluminescence immunoassay methods, (using Cobas e 411, Cobas e 601 and Cobas e 602, Roche^®^) (17). According to the employed method, anti-TPOAbs values above 34 IU/ml are defined as positive. TBAb antibodies (Thyroïd Receptor Binding antibodies) (18) were evaluated at baseline and at the hyperthyroid phase by electrochemiluminescence methods, using the system Cobas e801, Roche^®^. This a quantitative third generation TBAb method, in which patient's serum autoantibodies inhibit, *in vitro,* the fixation of a human monoclonal stimulating antibody (M22) against TSH Receptor (TSHR). According to the employed method, values above 1,2 IU/L are considered as positive.

Antiparietal cell antibodies research was based on indirect immunofluorescence methods (Menarini^®^). The screening dilution is 1/20. The results are reported as negative with a titer less than the screening dilution, positive with a titer greater than or equal to the screening dilution or, preferably, positive with specific endpoint titer.

Thyroid Ultrasounds examinations for assessment of echogenicity were always performed by experienced ultrasonographers (DB, HVS) with high–resolution machines. With regard to thyroid function patterns at diagnosis of both HT and GD, they were evaluated according to fT4 and TSH serum levels and classified into the following groups: 1) euthyroidism (both fT4 and TSH within normal limits); 2) hypothyroidism (low fT4, as opposed to elevated TSH) 3) overt hyperthyroidism (suppressed TSH, as opposed to elevated fT4) and 4) subclinical hyperthyroidism (supressed TSH, as opposed to normal fT4).

### Statistical analysis

Data were analysed using IBM SPSS statistics version 22 Software. The normality of the variables was estimated using the Shapiro test and Kolmogorov-Smirnov test. A descriptive statistics table of all variables was obtained, taking the median, range, mean and standard deviation among other parameters, as well as a table of frequencies.

## RESULTS

Twenty-four patients (22 women, 2 men) had hypothyroidism and HT followed by a phase of hyperthyroidism. They were followed up during a mean time of 48 ± 36 months. During hypothyroidism, mean levothyroxine treatment was 66.2 ± 30.8 µg/day. Following HT diagnosis, patients developed GD, after a mean time of 38 ± 45 months. None of the HT patients had initially features of Graves ophtalmopathy. Levothyroxine was stopped in all patients when hyperthyroidism was suspected. Suspicion of hyperthyroidism was raised because of frequent reduction of levothyroxine or the development of overt biological hyperthyroidism during follow up in some cases. The baseline characteristics and comparative descriptive statistics are shown in Table 1.

**Table 1:** Descriptive statistics of 24 cases of patients that developed Graves disease after Hashimoto Thyroiditis

	Hashimoto Thyroiditis hypothyroidism				Graves hyperthyroidism				
	Mean	SD	Median	Range	Mean	SD	Median	Range	p
Age at diagnosis (years)	39	13	39	18	42	11.3	42	45	ns
Levothyroxine dose (µg/day)	66	30	58.7	125	na	na	na	na	na
Thyroid volume (mL)	7.4	3.1	7.2	12.3	7.38	3.1	5.2	12	ns
TSH (mUI/L)	9.4	26.1	3	12.2	< 0.01	-	< 0.01	-	< 0.05
FT3 (pmol/L)	3.6	1.8		4.7	12	7.9	8.8	24	< 0.05
FT4 (pmol/L)	8.7	5.1	10.5	13.5	34	32	24.5	31	< 0.05
Anti TPO antibodies (IU/mL)	383	988	146	4735	335	240	273	580	ns
TBII (IU/L)	nd	nd	nd	nd	7.9	7.1	4.8	13	na
Switch from HT to GD (months)	na	na	na	na	38	45	18	168	na

nd: not done; ns: non significant; na: not applicable.

Patients developed hyperthyroidism with mean TRAb levels of 7.9 ± 7.1 IU/L. One hypothyroid patient developed hyperthyroidism three months *post-partum* and another hypothyroid patient was diagnosed with Graves disease during the first trimester of her four pregnancy. Hyperthyroidism was treated with radioiodine in 7 patients and with antithyroid drugs in 17 patients. Mean time to resolve hyperthyroidism in the radioiodine group was 6 ± 1 months whereas in the methimazole group it was 18 ± 2 months. After Graves disease, all patients were hypothyroid. They needed a mean levothyroxine treatment of 85 ± 20 µg/day.

During the hyperthyroid phase follow-up, four patients developed moderate Graves’ ophthalmopathy and three patients (10%) had anti-parietal cell antibodies. These three patients had a moderate gastritis pattern at gastric biopsy, without gastric atrophy. *Helicobacter pylori* were found in two of the three patients. As expected, levels of fT3, fT4 and TRAb were significantly higher and TSH levels was significantly lower at the hyperthyroid state (p < 0.05). There were no significant differences regarding the mean thyroid volume in hypothyroid nor in hyperthyroidism state.

## DISCUSSION

The conversion of autoimmune hypothyroidism to Graves’ hyperthyroidism is a rare clinical situation that is seldom described in text books and cases reports in the literature (1,13,19). This is one of the largest adult series of reported cases in which the conversion of hypothyroidism to hyperthyroidism was well documented (Table 2).

**Table 2:** Conversion from Hashimoto to Graves’ disease: reported series of adult patients

	Number of patients	Mean age	Time to develop hyperthyroidism from hypothyroidism	TSAb Positive (n cases)	TBAb Positive (n cases)	Mean Anti TPO (IU/mL)
Osorio-Salazar (19)^a^	7	33	36 months	Positive (hyper)	Negative (Hyper)	414
Takasu and cols.^b^ (21)	2	45	NA	110 (Hypo) 900 (Hyper)	98 (Hypo) 20 (Hyper)	NA
Takeda and cols. (23)	1	48	36 months	93 (Hypo) 163 (Hyper)	96 (Hypo) 31 (Hyper)	105
Cho and cols. (24)	1	40	12 months	92 (Hypo) 2703 (Hyper)	89 (Hypo) 12 (Hyper)	6400
Takasu and cols. (25)	8	38	NA	92 (Hypo) 1490 (Hyper)	96 (Hypo) 2 (Hyper)	800
Kraiem and cols. (26)	1	55	36 months	576 (Hyper)	Negative (Hyper)	NA
This series	24	36.1	38 months	Not done	24	228

All patients were treated with LT4.

Values for all thyroid autoantibodies are expressed in μU/mL; Neg, negative (undetectable); NA; not available.

a In this study TSAb and TBAb values were only registrated in two cases out of seven patients.

b Only two patients of 34 described by Takasu and cols. (21) had hypothyroidism due to TBAb and a switch to TSAb during hyperthyroidism.

Hyper: hyperthyroidism; hypo: hypothyroidism; TBAb: TSH blocking autoantibodies; TPOAb: autoantibodies to thyroid peroxidase; TSAb: thyroid-stimulating autoantibodies.

The switch from HT to GD depends on two TSH receptor binding antibodies with different effect in thyroid cells. Firstly, TSH receptor stimulating antibodies (TSAb) are responsible for Graves’ disease. Secondly, TSH receptor antibodies with inhibitory activity (TBAb) cause, unfrequently, a situation of hypothyroidism. The mechanism of TBAb hypothyroidism is different from the one caused by destructive Hashimoto's thyroiditis (8). The nomenclature for the methods used in the measurement of the antibodies against the TSH receptor is confusing. In short, TBII (TSAb and TBAb) antibodies measure the response of antibodies to TSHR, but they do not differentiate the functional activity (whether they are stimulatory, blocking or neutral). Instead, TSI antibodies measure functionality, as they stimulate TSHR receptor through the production of cAMP (12,20).

As we can see in Figure 1, depending on the affinity of the ligand, we can find in the same patient a predominance of the stimulatory or the blocking action of the receptor, thus resulting in hypo or hyperthyroidism (13).

One limiting factor of our study is the retrospective design. Another limiting factor is that TBAb were not systematically studied, as no apparent reason was present to dose these antibodies in hypothyroid patients. However, Takasu and cols studied TBAb and TSAb in 34 TBAb positive known patients with hypothyroidism. They demonstrated during follow up that with the disappearance of TBAb, there was recovery from hypothyroidism in 13 (87%) of these patients. Moreover, two of the 34 (5.9%) TBAb-positive patients with hypothyroidism developed TSAb-positive Graves’ hyperthyroidism (21). Osorio-Salazar and cols reported in 1994 a French series of seven females with HT who presented after a few months or years signs of Graves’ disease. Retrospectively, TSAb were found in some of the patients that switched form hypothyroidism into hyperthyroidism (19).

There are several mechanisms involved in the switching from TBAb to TSAb or *vice versa.* These include effects and circumstances that affect the autoimmune response, such as levothyroxine and antithyroid drug replacement therapy, immunosuppression/hemodilution occurring during pregnancy, and the disappearance of this postpartum immune tolerance and inherent properties of TSAb and TBAb (13,21). These authors studied whether there was a correlation between the dose of treatment with levothyroxine and the levels of anti-TPO antibodies with the development of TBII antibodies and subsequent hyperthyroidism. They found a significant association with anti-TPO antibodies levels, but no association with the dose of levothyroxine was found (13,21).

The effect of LT treatment on thyroid autoimmunity is an old ‘workhorse’ with countless reports in the literature for more than 50 years (8). Recent longitudinal studies suggest that the effect of LT treatment has little or no effect on anti-TPO and anti-thyroglobulin antibodies in euthyroid patients or in patients with subclinical hypothyroidism. The effect of LT on hypothyroid patients is more debated. Takasu and Matshushita's studies have reported a positive correlation between LT dose and the appearance of *de novo* TSAb autoantibodies or increased TSAb antibody levels (21). They also described the development of hyperthyroidism in hypothyroid patients. This association may simply be fortuitous or it can be explained by the regulatory effect of thyroid hormones on the innate and adaptive immune response: Dendritic cells modulate their phenotype under the influence of elevated levels of thyroid hormone (27).

Another possible explanation for this transition from hypothyroidism to hyperthyroidism (21-28) is that the autoimmune tissue damage (initially severe enough to cause thyroid hypofunction), recovers sufficiently to allow subsequent stimulation by TSAb (28). This expansion of the autoimmune response has been called ‘determinant spreading’, where a large number of different T cell clones attack a broader range of determinants of the invading pathogens or the inflamed tissues, instead of focusing on few immunodominant determinants (29,30). In this sense, it seems that longstanding hypothyroidism leads to increased and permanent tissue damage and there is likely less functioning thyroid tissue able to react to thyrotropin receptor autoantibodies as compared to hypothyroidism of short evolution (5,29,30).

Finally, we detected a thyroid and a gastric autoimmunity in three of 14 (21%) patients. Asymptomatic autoimmune gastritis is prevalent in patients with autoimmune thyroiditis as we and other have already reported (31-33). The interest of diagnosing autoimmune gastritis in these patients is to evaluate the possible impact of hypoclorydia in levothyroxine and micronutriments (iron, vitamin B12) malabsorption. In two of our patients, *Helicobacter pilory* was found in gastric biopsies, suggesting its role in gastric autoimmunity. Moreover, a possible effect of *Helicobacter pilory* infection has been suggested in thyroid autoimmunity because of cross autoantigens reaction between the bacteria capsid and gastric cells (31-33).

In conclusion, conversion of Hashimoto Thyroiditis towards Graves’ disease is still an underestimated clinical feature for most clinicians. In our series, this phenomenon was observed in 1.2% of patients. Interestingly, the conversion from HT to GD has been described as prevalent (25.7% of cases) in a controlled series of 35 children with either Down or Turner Syndrome (33). Our data, as well as previous reported cases should raise the suspicious of this phenomenon whenever hyperthyroidism and difficulties in equilibrating patients are observed in “usual” autoimmune hypothyroid patients supplemented with levothyroxine. Several mechanisms have been postulated behind the conversion from HT to GD. One of them is the presence of different autoantibodies that include thyroid stimulating antibodies and thyroid blocking antibodies. Another possible explanation for this transition is that the autoimmune tissue damage, initially severe enough to cause thyroid hypofunction, recovers sufficiently to allow subsequent stimulation by TSAb. More extensive cohort immunological and genetic studies are necessary to gain insight into these interesting observations.
